# Xpert Ultra on nasopharyngeal swabs detects one-third of pulmonary tuberculosis: a nested case-control study in South African primary care

**DOI:** 10.21203/rs.3.rs-10112219/v1

**Published:** 2026-06-23

**Authors:** Robinah Nakawunde, Jay Achar, Brian Allwood, Suventha Moodley, Loren Rockman, Anna Okunola, Daphne Naidoo, Niaz Banaei, Charissa C. Marsh, Grant Theron

**Affiliations:** Stellenbosch University; Stellenbosch University; Tygerberg Hospital; Stellenbosch University; Stellenbosch University; Stellenbosch University; Stellenbosch University; Stanford University School of Medicine; Stellenbosch University; Stellenbosch University

**Keywords:** Nasopharyngeal swabs, tongue swabs, tuberculosis, Xpert MTB/RIF Ultra

## Abstract

**Background:**

Sputum-free alternatives for tuberculosis (TB) diagnosis are a public health priority. Nasopharyngeal swabs (NPSs), a specimen routinely used for viral testing especially at-scale in outbreak investigations, may have promise but has not been evaluated in people investigated for TB.

**Methods:**

We evaluated Xpert MTB/RIF Ultra (Ultra; Cepheid, Sunnyvale, California, USA) on stored NPSs (NPSU) from adults with presumptive TB consecutively presenting to primary care in Cape Town, South Africa. Using a nested case-control design, all participants with TB had a NPS tested (n = 148) and people without TB (n = 117) were randomly selected matched by HIV status. We used a microbiological (MRS) and extended microbiological reference standards (eMRS). A subset of convenience-sampled casescontrols also received flocked tongue swab Ultra (TSU) (n = 57 cases, n = 66 controls).

**Results:**

Twenty-one (8%) of NPSUs were unsuccessful (all errors). NPSU sensitivity was 35% (95% CI 27, 43), specificity 88% (80, 93). Sensitivity was lower in HIV-positive participants [24% (14, 36) vs. 44% (32, 56)]. TSU had higher sensitivity [67% (53, 79) vs. 28% (17, 42) in the head-to-head comparison, detecting 22 additional cases] than NPSU. Specificity was comparable.

**Conclusion:**

NPSU sensitivity (35%) was lower than TSU and below the WHO target of 80% for non-sputum confirmatory tests; specificity (88%) was also below the 98% target. NPSs may have utility if already collected in mass testing campaigns for other diseases but unless performance can be optimized (e.g., by using purpose-built swab platforms) do not appear useful for TB diagnosis.

## Background

Tuberculosis (TB) is the leading infectious cause of mortality, with 10.7 million incident cases and 1.23 million deaths in 2024^1^. Despite progress in diagnostic technologies, 22% of TB cases are undiagnosed or unreported, and only 54% of those notified were initially tested with a World Health Organization (WHO)-recommended molecular test^1^. This persistent diagnostic gap impedes progress towards the End-TB targets^2^. To address it, especially in people living with HIV (PLHIV) who often present with paucibacillary disease or inability to produce sputum^3–5^, non-sputum-based tests are a priority^6^.

Molecular tests like Xpert MTB/RIF Ultra (Ultra; Cepheid, Sunnyvale, California, USA) improved TB detection^7,8^. Given that *Mycobacterium tuberculosis* complex (MTBC) infection occurs primarily through inhalation of aerosolized droplet nuclei that deposit along the respiratory tract^9^, the nasopharyngeal mucosa represents an anatomically plausible sampling site for diagnosis. This is further supported by a recent microbiome study of the respiratory tract in pulmonary TB, which found that, after the lungs, the nasopharynx had the highest relative abundance of mycobacterial DNA^10^.

Nasopharyngeal swabs (NPSs) are routinely used for detection of respiratory pathogens and, as demonstrated during COVID-19^11–13^, offer practical advantages, such as ease-of-collection, patient acceptability, scalability, and easy shipping and transport. If used for TB, NPSs could expand testing in those unable to produce sputum, enhancing diagnostic yield^14,15^. In case of future pandemics when NPSs are collected anyway for respiratory pathogen diagnostic testing, concurrent testing for TB could occur, mitigating stagnations in TB testing like those witnessed during COVID-19^16,17^.

To our knowledge, NPSs have not been evaluated for diagnosis in adults undergoing routine investigation for TB. One study assessing Ultra on NPSs (NPSU) was conducted postmortem and reported 70% (50, 86) sensitivity and 99% (96, 100) specificity^7^. This highlighted their potential; however, bacillary distribution and burden in postmortem specimens may differ from those in ambulatory patients. We therefore evaluated the diagnostic accuracy and yield of NPSU in adults with presumptive pulmonary TB attending primary care. As tongue swabs (TSs) were recently endorsed by the WHO for TB testing^18^ we also, using previously reported TS Ultra (TSU) performance data in a subset of the same participants, compared performance with NPSU.

## Methods

### Study cohort

Adults (≥ 18 years) self-reporting with symptoms meeting WHO symptom criteria for presumptive pulmonary TB^19^ (n = 732) were recruited from primary healthcare facilities in Cape Town, South Africa between September 2020 and July 2023. Sociodemographic, clinical, and microbiological data were recorded.

### Sample size

**Figure 1:**
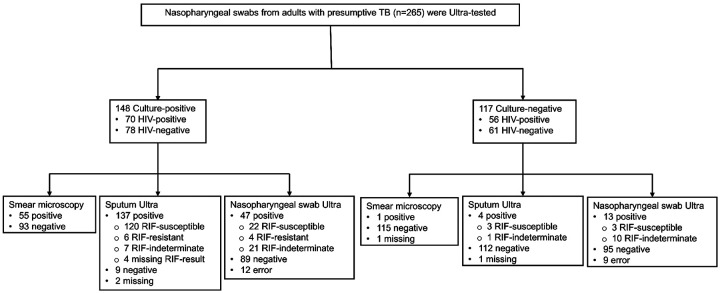
Study profile. NPSU was done for 265 participants (148 TB-positive and 117 TB-negative). Sputum smear microscopy, culture, and Ultra results were retrieved from the parent study database. Abbreviations: NPSU: nasopharyngeal swab Ultra, RIF: Rifampicin.

We used guidance for the evaluation of confirmatory TB tests^20^. After a pilot (n = 65; 34 TB-positive), NPSU had 44% sensitivity and 74% specificity against sputum culture. Using these point estimates with a target 95% CI total width of 20%, we calculated a required sample size of 265 participants (148 with TB, 117 without TB). This required testing all available NPSs from people with TB. In those without TB, we randomly selected a similar proportion of HIV-positive and HIV-negative participants as in those with TB (Fig. 1).

### Specimen collection

Each participant provided two sputa (induction available if need^21^) and one NPS in no fixed order. One sputum was processed for culture using the BACTEC MGIT (Mycobacteria Growth Indicator Tube) 960 system (Becton Dickinson, Sparks, Maryland, USA; with positive growth speciated using Ultra), and the other sputum tested using Ultra (SU) as recommended^22^. NPSs were collected by trained study nurses according to a standardised operating procedure and stored at − 20°C until testing [median (IQR) 14 months (10, 17)]. eNAT swabs (wet swabs; Copan, Brescia, Italy) were used for the first 145 participants (these collection tubes contained 2 mL of guanidine thiocyanate) after which dry swabs were adopted. This was based on limited availability of wet swabs and guidance that, for TSs, dry swabs were preferred to wet swabs^23^. 46% of NPSU-tested participants (123/265) had TSU data available, these participants were consecutively recruited and specimens collected and tested as described before^24^.

### NPS processing and Ultra testing

NPSs were thawed at room temperature and heat inactivated (100°C, 10 min). For wet swabs, the swabcontaining collection tube was vortexed (17.8 × *g*, 30 s), after which 1.5 mL of the suspension was mixed with 0.5 mL of Tris (10 mM, pH 8) EDTA (1 mM) (TE) buffer (HEPES Detergent, Cape Town, South Africa). The resulting 2 mL was transferred into an Ultra cartridge and thereafter processed as per manufacturer’s recommendation^22^. For dry swabs, 2 mL of TE was added following heat inactivation, vortexed (17.8 × *g*, 30 s), and the entire volume was loaded into the cartridge. No Ultra Sample Reagent (Cepheid) was used.

### Definitions

The microbiological reference standard (MRS) was positive if the MGIT960 culture had MTBC-positive growth (negative if no growth). The extended microbiological reference standard (eMRS) was positive if MRS-positive or SU-positive and negative if both culture and Ultra were negative. Unsuccessful results were those neither positive nor negative. Diagnostic yield among all those tested (DYT) was the proportion of all participants tested in whom NPSU was positive^14^.

### Analysis

Methods and reporting are per Standard for Reporting Diagnostic Accuracy (STARD) guidelines^25,26^ (**Table S3**).

#### Baseline characteristics

Count data were summarised as proportions, continuous variables as medians with interquartile ranges (IQR). Differences between groups were assessed using the chi-square test for categorical variables and the Mann-Whitney U test for continuous variables. Morbidity score information (TBscore II) was calculated from seven clinical variables (cough, haemoptysis, chest pain, night sweats, pale conjunctivae, body mass index (BMI), and mid-upper arm circumference). Higher scores indicate higher morbidity^27^.

#### Primary analysis

NPSU Sensitivity and specificity were estimated against the MRS and eMRS for the overall cohort and stratified by HIV status, sputum smear status, and previous TB history, with MRS results presented unless otherwise stated. DYT was calculated as Broger et al.^14^. Ultra sample processing control (SPC) cycle threshold (C_T_) values were compared between NPS and sputum specimens to assess assay inhibition, with higher SPC C_T_ values (> 34) indicating inhibition^28^. Ultra semiquantitative categories were reported as described in literature^8^.

#### Secondary analyses

Performance of wet versus dry NPSs was also compared, alongside head-to-head comparisons of NPSU, SU and TSU within the same participants. UpSet plots were generated using *ComplexUpset*^29,30^, version 1.3.6 in R version 4.5.1 (R Foundation for Statistical Computing, Vienna, Austria).

Diagnostic metrics were calculated using Excel software (Microsoft 365, version 2605, Redmond,Washington, USA) and compared using prtest^31^ and Fisher exact test in Stata software version 19.0 (StataCorp, College Station, Texas, USA), confidence intervals were computed using Wilson score intervals. Two-sided p values ≤ 0.05 were considered statistically significant.

### Ethics

The study was approved by the Stellenbosch University Health Research Ethics Committee (M21/10/022, M22/02/004_Sub Study M20/06/017) and the Western Cape Department of Health (WC_202106_055, WC_202203_035). Written informed consent was obtained from all participants.

## Results

### Participant characteristics

Of the 265 participants (148 with TB, 117 without TB), 118 (45%) were female and 95 (36%) had previous TB. MRS-positive participants were younger, more likely to be male, and had a lower BMI, vs those who were MRS-negative. Among PLHIV, MRS-positivity was more common in those not receiving antiretroviral therapy (ART) than those on ART (Table 1). 4/265 (2%) required induction to make sputum.

### Unsuccessful nasopharyngeal swab Xpert MTB/RIF Ultra results

Eight percent (21/265) of NPSUs were unsuccessful (all “error”), 12/21 (57%) from MRS-positive participants.

Performance of nasopharyngeal swab Xpert MTB/RIF Ultra did not meet WHO benchmarks.

#### Sensitivity and specificity

**Figure 2:**
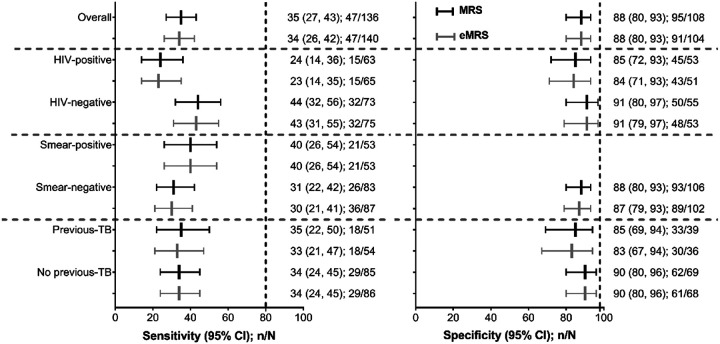
NPSU sensitivity (35%) and specificity did not meet WHO benchmarks. Sensitivity and specificity estimates are presented for MRS and eMRS in the overall cohort and clinically relevant stratifications. Vertical line at 80% sensitivity and 98% specificity show the respective minimum WHO performance standards for non-sputum confirmatory tests. Abbreviations: eMRS: extended microbiological reference standard, MRS: microbiological reference standard, WHO: World Health Organization.

Sensitivity was 35% (95% CI 27, 43) and specificity 88% (80, 93; Fig. 2). Sensitivity was lower in PLHIV versus HIV-negative people [24% (14, 36) vs. 44% (32, 56); p = 0.014]. Sensitivity did not differ by sputum smear status [40% (26, 54) in smear-positives vs. 31% in smear-negatives (22, 42); p = 0.321]. Specificity did not differ by previous TB history [85% (69, 94) vs. 90% in those without previous TB (80, 96); p = 0.422]. Performance against the eMRS was similar (Fig. 2). DYT was 23% (60/265).

#### False-negative and -positive results

**Figure 3:**
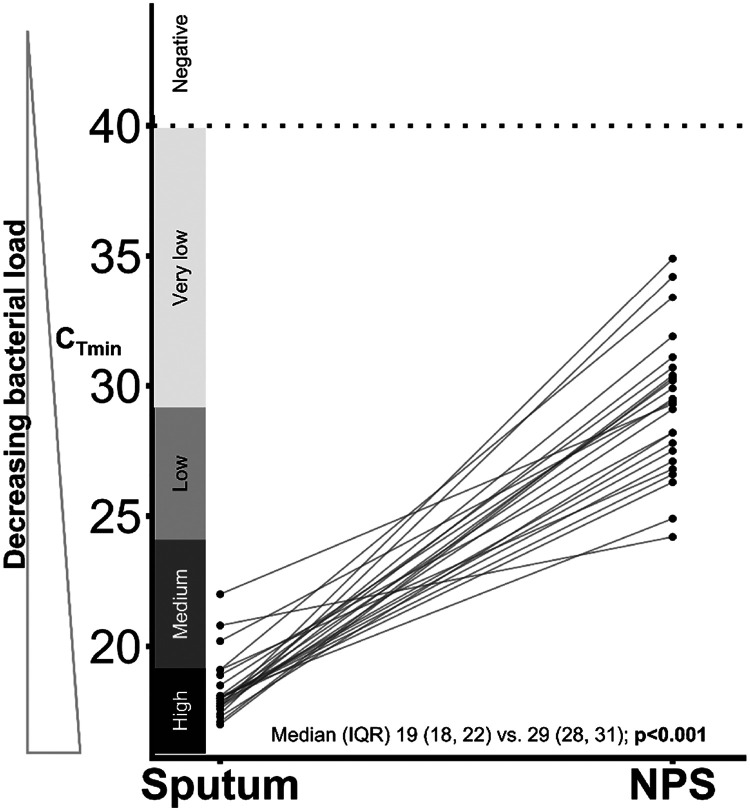
NPSU cycle threshold values were higher than those of paired sputum. Horizontal lines connect individual participant’s specimens. Lower C_T_ values correspond to higher bacillary load. Gray-scale bands represent Ultra semi-quantitative categories. The dotted line at C_T_=40 shows the assay positivity threshold. Abbreviations: C_Tmin_: minimum cycle threshold for rpoB readout, NPS: nasopharyngeal swab.

Of the 136 MRS-positive participants with valid NPSU result, 89 (65%) had false-negative NPSU results, more common in PLHIV and those with lower bacillary load (longer sputum-culture time-to-positivity; Table 2). NPSU was false-positive in 13/108 (12%) of participants, 10/13 (77%) semi-quantified as “trace” and 3/13 (23%) as “very low”. Among these, 8/13 (62%) were PLHIV, 6/13 (46%) had previous TB and sputum Ultra was negative in all. Compared with paired sputum, NPSs had lower bacillary burden, reflected by higher Ultra C_T_ values [29 (28, 31) vs. 19 (18, 22); p < 0.001] (Fig. 3). Inhibition was detected in 3 (1%) of SUs and no NPSUs.

#### Wet and dry swabs

Wet NPSUs showed higher sensitivity than dry NPSUs [45% (34, 57) vs. 21% (12, 34); p = 0.003], and similar specificity [90% (79, 97) vs. 86% (74, 94); p = 0.499]. Wet NPSU sensitivity did not differ by HIV status [42 (25, 61) in PLHIV vs. 48 (32, 63) HIV-negative people; p = 0.620], whereas dry NPSU sensitivity was lower in PLHIV [6% (1, 21) vs. 38% (21, 58); p = 0.003; **Table S1**]. We evaluated if differences in sputum bacterial load between people who gave a wet or dry swab may account for sensitivity differences however, among MRS-positive participants, median (IQR) culture time-to-positivity was similar between swab types [9 (6, 13) for wet vs. 8 days (6, 12) for dry; p = 0.582].

Tongue swabs were more sensitive than nasopharyngeal swabs.

**Figure 4:**
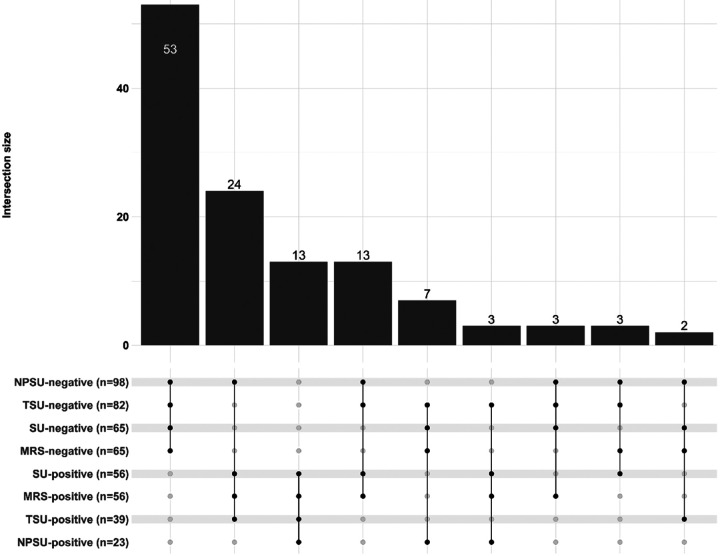
TSU identified more culture-positive TB cases than NPSU. UpSet plot shows overlap of results from sputum culture, SU, NPSU and TSU among 121 participants with valid results for all tests. Bar heights show the number of participants within each intersection, while connected black dots identify the combination of tests contributing to that intersection. Notably, twenty-four participants were MRS-, SU-, and TSU-positive but NPSU-negative. Abbreviations: MRS: microbiological reference standard, NPSU: nasopharyngeal swab Ultra, SU: sputum Ultra, TSU: tongue swab Ultra.

TSU results were available for 123/265 (46%) of participants. Characteristics were similar in participants with or without TSU, with the people previously tested using TSU having a low culture-positive rate **(Table S2)**. TSU sensitivity was higher than NPSU in this subset [67% (53, 79) vs. 28% (17, 42); p < 0.001], while specificity was comparable [97% (89, 100) vs. 89% (79, 96); p = 0.459]. Similar findings were observed across HIV status, smear status, and previous TB strata (Table 3). NPSU missed 24 cases identified by both SU and TSU (Fig. 4).

## Discussion

We showed 1) NPSU sensitivity was low (35%) and specificity moderate (88%; improving with 97% with reclassification of trace results), with neither meeting the WHO minimum benchmark for low complexity non-sputum confirmatory tests (80% sensitivity, 98% specificity)^6^; 2) despite prior reports of high mycobacterial read counts from NPs, NPSU results had lower sensitivity than SU as well as 3) TSU, which is less invasive. Lastly, 4) use of wet swabs was associated with higher NPSU sensitivity than dry swabs. Together, these data suggest NPSs are not valuable for TB diagnosis in our population.

NPSU sensitivity (35%) was similar to Ultra on other non-sputum specimens, including urine (32%)^32^ and blood (37%)^33^ in PLHIV and comparable to pharyngeal swabs in HIV-negative adults (38%)^34^. Although lower than the previously reported NPSU sensitivity in decedents (70%)^7^, the ambulatory cohort in our study better reflects real-world conditions, providing a pragmatic estimate of NPSU performance in routine care.

NPSU sensitivity was lower in PLHIV versus those without HIV, consistent with Ultra on other specimen types^35^. The magnitude of this sensitivity reduction exceeded that reported for sputum Ultra^36,37^, suggesting a greater HIV-associated bacillary reduction in the nasopharynx than in sputum. Specificity did not differ by prior TB history, unlike Ultra on sputum^36,37^, possibly as prior TB in this cohort was remote (~ 6 years). Although most NPSU results with a “trace” semi-quantitation category were true-positive, false-positives were themselves predominantly “trace”, occurring more frequently in PLHIV. Such false-positives represent low-level early active TB, transient upper-airway colonisation, or residual nonviable DNA. Supporting the first possibility, a prospective study in Chile found most NPSU-positive TB contacts were later diagnosed with active TB^38^. Overall, trace-positive NPSU results should be interpreted cautiously.

Ultra positivity and bacillary load were lower in NPSs than sputum, suggesting the nasopharynx harbours less MTBC than sputum with more individuals below Ultra’s limit of detection^8,39^. Other contributors to lower sensitivity may include specimen dilution, small specimen volumes, and matrix effects that hamper the release of material from the swab. This may explain why purpose-built swab molecular assays, such as MiniDock MTB (Pluslife, China), outperform off-label use of sputum-optimised platforms like Ultra on TSs^40^. Such platforms warrant evaluation with NPSs.

TSU had higher sensitivity than NPSU with comparable specificity, with TSU performance consistent by HIV status, supporting TSU as the preferred specimen to NPSU. These findings align with emerging evidence for oral sampling in high burden settings^35,41^ and WHO endorsement of TSs as simple, accessible TB specimens^18^, with additional advantages in acceptability, affordability and comfort^42,43^.

NPSU on wet swabs had higher sensitivity than on dry swabs, particularly among PLHIV. This may reflect guanidine thiocyanate stabilising nucleic acids and improving downstream molecular detection after storage. The similar sputum culture time-to-positivity across groups suggests performance differences were unlikely due to bacterial burden differences. Together, these findings suggest preferential use of wet swabs, especially in PLHIV, should NPSU testing be pursued and especially if dry swab testing cannot be done promptly.

Our study had strengths and limitations. Strengths included use an eMRS, consecutive enrolment from primary care, balanced HIV representation, and comparisons with tongue swab and sputum Ultra. Limitations include a lack of direct head-to-head comparisons (wet vs dry NPSs, NPSs vs TSs) and retrospective NPS testing (which may be partly responsible for sensitivity differences in wet vs dry NPSs). Prospective testing of dry swabs may therefore generate sensitivity estimates closer to those obtained retrospectively using wet swabs. Most of our population could expectorate sputum, limiting relevance for sputum-scarce people who tend to have milder disease^5^. Finally, the nested case-control design may limit generalisability of specificity estimates, as controls may not fully reflect the spectrum of disease seen in routine practice.

In conclusion, although NPSU did not meet WHO target product profile benchmarks for a stand-alone diagnostic test, NPSs may still warrant further investigation if already collected during mass testing campaigns for other diseases, however, purpose-built swab-based TB test platforms should be used prospectively. This future work should also focus on populations in which sputum expectoration is challenging.

## Supplementary Material

Supplementary Files

This is a list of supplementary files associated with this preprint. Click to download.

- Supplementaryfile.docx

## Figures and Tables

**Table 1.** Baseline demographics and clinical characteristics. Participants with TB were more likely to be men, be younger or have lower BMI, and, among those with HIV, likely to not be on ART. Data are median (IQR) or n (%).

Characteristics	Overall n=265	MRS-negative n=117	MRS-positive n=148	p-value
*Demographics*				
Age, years	37 (28, 47)	40 (31, 48)	36 (28, 43)	**0.033**
Female	118 (45)	63 (54)	55 (37)	**0.007**
BMI (kg/m^2^)	19.9 (18.2, 23.7)	22.3 (19.0, 27.5)	19.2 (17.7, 21.4)	**<0.001**
*Clinical*				
TBscore II	2 (2, 3)	2 (2, 3)	3 (2, 4)	**<0.001**
PLHIV	126 (48)	56 (48)	70 (47)	0.927
on ART	65 (52)	42 (75)	23 (33)	**<0.001**
Previous TB	95 (36)	41 (35)	54 (36)	0.808
Years since prior TB	7 (4, 13)	9 (5, 15)	7 (4, 11)	0.142

p-values compare characteristics of MRS-negative vs. MRS-positive, bold font denotes p-values ≤0.05.

Missing data: Interval since prior TB (n=35).

Abbreviations: ART: antiretroviral therapy, BMI: body mass index, IQR: interquartile range, PLHIV: people living with HIV, TBscore II: tuberculosis symptom score II^27^.

**Table 2.** Factors associated with false-negative NPSU results among MRS-positive participants. False-negative NPSU results were more common among PLHIV and those with lower bacillary load, reflected by longer sputum culture time-to-positivity. Data are median (IQR) or n (%).

Characteristics	True-positive n=47	False-negative n=89	p-value
*Demographics*			
Age, years	33 (26, 40)	36 (28, 48)	**0.046**
Female	14 (30)	38 (43)	0.141
BMI (kg/m^2^)	18.8 (17.3, 20.7)	19.3 (17.7, 22.0)	0.158
*Clinical*			
Previous TB	18 (36)	33 (37)	0.889
TBscore II	3 (2, 4)	3 (2, 4)	0.875
PLHIV	15 (32)	48 (54)	**0.014**
On ART	5 (33)	14 (29)	0.757
Culture time to positivity	7 (5, 10)	10 (7, 13)	**0.016**

p-values compare true-positive vs. false-negative, bold font denotes p-values ≤0.05.

Abbreviations: ART: antiretroviral therapy, BMI: body mass index, IQR: interquartile range, PLHIV: people living with HIV, TBscore II: tuberculosis symptom score II^27^.

**Table 3.** Ultra performance on NPSs compared with TSs. TSU-sensitivity was higher than that of NPSU but specificity did not differ. TSU showed higher sensitivity than NPSU, while specificity was comparable between specimens. Data are % (95% CI); n/N.

	Sensitivity		Specificity	
	NPSU	TSU	NPSU	TSU
Overall (n=123)	28 (17, 42); 16/57	67 (53, 79); 38/57	89 (79, 96); 59/66	97 (89, 100); 64/66
		^†^ **p<0.001**		^†^p=0.084
HIV-positive (n=58)	20 (7, 41); 5/25	52 (31, 72); 13/25	85 (68, 95); 28/33	97 (84, 100); 32/33
		^†^ **p=0.018**		^†^p=0.087
HIV-negative (n=65)	34 (19, 53); 11/32	78 (60, 91); 25/32	94 (80, 99); 31/33	97 (84, 100); 32/33
		^†^ **p<0.001**		^†^p=0.555
	^‡^p=0.231	^‡^ **p=0.038**	^‡^p=0.087	^‡^p>0.999
Smear-positive (n=14)	36 (13, 65); 5/14	93 (66, 100); 13/14		
		^†^ **p=0.002**		
Smear-negative (n=108)	26 (14, 41); 11/43	58 (42, 73); 25/43	89 (79, 99); 58/65	97 (89, 100); 63/65
		^†^ **p=0.002**		
	^‡^p=0.464	^‡^ **p=0.017**		^†^p=0.084
Previous TB (n=46)	30 (13, 53); 7/23	65 (43, 84); 15/23	91 (72, 99); 21/23	100 (85, 100); 23/23
		^†^ **p=0.018**		^†^p=0.148
No previous TB (n=77)	26 (13, 44); 9/34	68 (49, 83); 23/34	88 (75, 96); 38/43	95 (84, 99); 41/43
		^†^ **p<0.001**		^†^p=0.237
	^‡^p=0.744	^‡^p=0.599	^‡^p=0.712	^‡^p=0.294

Specificity could not be computed for the smear-positive subgroup because all participants had a positive reference standard result.

† Within row p-values: NPSU vs. TSU.

‡ Within column p-values: HIV-negative vs. HIV-positive, sputum smear-positive vs. smear-negative and previous TB vs. no previous TB. Bold font denotes p≤0.05.

Abbreviations: MRS: microbiological reference standard, NPSU: nasopharyngeal swab Ultra, TSU: tongue swab Ultra, TB: tuberculosis.

## Data Availability

Data will be made available upon request directly from the corresponding authors and will include de-identified participant data and data dictionaries. Data requests will be reviewed and examined by the corresponding author and other study participants. The ethical and legal implications of data sharing will be examined. Depending on how the review turns out, data will be disclosed.

## Abbreviations

ART: antiretroviral therapy
BMI: body mass index
IQR: interquartile range
PLHIV: people living with HIV
TBscore: Tuberculosis symptom score ||^27^

## References

[1] World Health Organization (2025) Global Tuberculosis Report

[2] World Health Organization (2015) THE END TB STRATEGY

[3] World Health Organization (2019) Lateral flow urine lipoarabinomannan assay (LF-LAM) for the diagnosis of active tuberculosis in people living with HIV: policy update 2019

[4] Vargas D, García L, Gilman RH (2005) Diagnosis of sputum-scarce HIV-associated pulmonary tuberculosis in Lima, Peru. Lancet 365(9454):150–152 15639297 10.1016/S0140-6736(05)17705-8 PMC2912523

[5] Nwamba WV, Okunola AO, Crowder R (2026) Sputum scarcity and associated factors in people undergoing tuberculosis testing in South Africa, Uganda, India, and the Philippines: an analysis of cross-sectional observational data. EClinicalMedicine; 95 10.1016/j.eclinm.2026.103977 PMC13195755 42180402

[6] Kohli M, Korobitsyn A, Ismail N (2025) WHO target product profile for TB detection at peripheral settings: 2024 update. PLOS global public health 5(6):e0004612 40498791 10.1371/journal.pgph.0004612 PMC12157113

[7] Costales C, Crump JA, Mremi AR (2022) Performance of Xpert Ultra nasopharyngeal swab for identification of tuberculosis deaths in northern Tanzania. Clin Microbiol Infect 28(8):1150e1–.e6 10.1016/j.cmi.2022.03.027 PMC11566265 35358686

[8] Chakravorty S, Simmons AM, Rowneki M (2017) The New Xpert MTB/RIF Ultra: Improving Detection of Mycobacterium tuberculosis and Resistance to Rifampin in an Assay Suitable for Pointof-Care Testing. mBio; 8(4) 10.1128/mBio.00812-17 PMC5574709 28851844

[9] Coleman M, Martinez L, Theron G, Wood R, Marais B (2022) Mycobacterium tuberculosis transmission in high-incidence settings—new paradigms and insights. Pathogens 11(11):1228 36364978 10.3390/pathogens11111228 PMC9695830

[10] Chiyaka TL, Moodley S, Simon D (2026) Bacterial topography of the respiratory tract, including pulmonary site-of-disease, in people with active tuberculosis: a case-control study. Res Square. 10.21203/rs.3.rs-9956587/v1

[11] Thomas HM, Mullane MJ, Ang S (2022) Acceptability of OP/Na swabbing for SARS-CoV-2: a prospective observational cohort surveillance study in Western Australian schools. BMJ open 12(1):e055217 10.1136/bmjopen-2021-055217 PMC8808315 35082134

[12] Møller IJB, Utke AR, Rysgaard UK, Østergaard LJ, Jespersen S (2022) Diagnostic performance, user acceptability, and safety of unsupervised SARS-CoV-2 rapid antigen-detecting tests performed at home. Int J Infect Dis 116:358–364 35038598 10.1016/j.ijid.2022.01.019 PMC8759098

[13] Valentine-Graves M, Hall E, Guest JL (2020) At-home self-collection of saliva, oropharyngeal swabs and dried blood spots for SARS-CoV-2 diagnosis and serology: Post-collection acceptability of specimen collection process and patient confidence in specimens. PLoS ONE 15(8):e0236775 32756585 10.1371/journal.pone.0236775 PMC7406082

[14] Broger T, Marx FM, Theron G (2024) Diagnostic yield as an important metric for the evaluation of novel tuberculosis tests: rationale and guidance for future research. Lancet Global Health 12(7):e1184–e91 38876764 10.1016/S2214-109X(24)00148-7

[15] de Nooy A, Ockhuisen T, Korobitsyn A (2024) Trade-offs between clinical performance and test accessibility in tuberculosis diagnosis: a multi-country modelling approach for target product profile development. Lancet Global Health 12(7):e1139–e48 38876761 10.1016/S2214-109X(24)00178-5

[16] McQuaid CF, Vassall A, Cohen T, Fiekert K, White R (2021) The impact of COVID-19 on TB: a review of the data. Int J Tuberc Lung Dis 25(6):436–446 34049605 10.5588/ijtld.21.0148 PMC8171247

[17] World Health Organization (2022) Global Tuberculosis Report

[18] World Health Organization. Near point-of-care tests, tongue swabs, and sputum pooling for TB (2026) https://www.who.int/teams/global-programme-on-tuberculosis-and-lung-health/diagnosis-treatment/npoc-tongue-swabs-and-sputum-pooling-for-tb (accessed May-12 2026)

[19] World Health Organization (2021) Operational handbook on tuberculosis: module 2: screening: systematic screening for tuberculosis disease 33822560

[20] Schumacher SG, Wells WA, Nicol MP (2019) Guidance for studies evaluating the accuracy of sputum-based tests to diagnose tuberculosis. J Infect Dis 220(Supplement3):S99–S107 31593597 10.1093/infdis/jiz258 PMC6782025

[21] Peter JG, Theron G, Singh N, Singh A, Dheda K (2014) Sputum induction to aid diagnosis of smear-negative or sputum-scarce tuberculosis in adults in HIV-endemic settings. Eur Respir J 43(1):185–194 23520317 10.1183/09031936.00198012 PMC5454491

[22] Cepheid Xpert MTB/RIF Ultra Product Brochure. 2022–2023

[23] Andama A, Steadman AE, Ahls C (2024) Consensus standard operating procedure for collection of tongue swabs for TB diagnostics. protocolsio part Springer Nat

[24] Ajide B, Moe CA, Barrameda J (2026) Tongue swab Xpert MTB/RIF Ultra testing for TB using a revised consensus protocol. Int J Tuberc Lung Dis 30(5):211–216 42046222 10.5588/ijtld.25.0520

[25] Cohen JF, Korevaar DA, Altman DG (2016) STARD 2015 guidelines for reporting diagnostic accuracy studies: explanation and elaboration. BMJ open 6(11):e012799 10.1136/bmjopen-2016-012799 PMC5128957 28137831

[26] Bossuyt PM, Reitsma JB, Bruns DE (2015) STARD 2015: an updated list of essential items for reporting diagnostic accuracy studies. Radiology 277(3):826–832 26509226 10.1148/radiol.2015151516

[27] Rudolf F, Lemvik G, Abate E (2013) TBscore II: refining and validating a simple clinical score for treatment monitoring of patients with pulmonary tuberculosis. Scand J Infect Dis 45(11):825–836 24041274 10.3109/00365548.2013.826876

[28] Blakemore R, Nabeta P, Davidow AL (2011) A multisite assessment of the quantitative capabilities of the Xpert MTB/RIF assay. Am J Respir Crit Care Med 184(9):1076–1084 21836139 10.1164/rccm.201103-0536OC PMC3208646

[29] Lex A, Gehlenborg N, Strobelt H, Vuillemot R, Pfister H (2014) UpSet: visualization of intersecting sets. IEEE Trans Vis Comput Graph 20(12):1983–1992 26356912 10.1109/TVCG.2014.2346248 PMC4720993

[30] Krassowski M (2025) *complexUpSet*: Create Complex UpSet Plots Using ggplot2 Components. R package version 1.3.6. https://github.com/krassowski/complex-upset

[31] StataCorp (2026) prtest—tests of proportions. https://www.stata.com/manuals/rprtest.pdf

[32] Sossen B, Székely R, Mukoka M (2024) Urine-Xpert Ultra for the diagnosis of tuberculosis in people living with HIV: a prospective, multicentre, diagnostic accuracy study. Lancet Global Health 12(12):e2024–e2034 39577975 10.1016/S2214-109X(24)00357-7 PMC11584317

[33] Boloko L, Schutz C, Sibiya N (2022) Xpert Ultra testing of blood in severe HIV-associated tuberculosis to detect and measure < em>Mycobacterium tuberculosis blood stream infection: a diagnostic and disease biomarker cohort study. Lancet Microbe 3(7):e521–e32 35644157 10.1016/S2666-5247(22)00062-3 PMC9242865

[34] Liu Y, Hu Q, Guo S (2025) Comparison of diagnostic efficacy of three different oral samples in pulmonary tuberculosis using cepheid gene Xpert^®^ MTB/RIF ultra. J Microbiol Methods : 107310 41176289 10.1016/j.mimet.2025.107310

[35] Rockman L, Abdulgader SM, Minnies S (2025) Oral Washes and Tongue Swabs for Xpert MTB/RIF Ultra–Based Tuberculosis Diagnosis in People With and Without the Ability to Make Sputum. Clin Infect Dis 81(6):e632–e42 10.1093/cid/ciaf397 40686067

[36] Theron G, Venter R, Calligaro G (2016) Xpert MTB/RIF Results in Patients With Previous Tuberculosis: Can We Distinguish True From False Positive Results? Clin Infect Dis 62(8):995–1001 26908793 10.1093/cid/civ1223 PMC4803105

[37] Theron G, Venter R, Smith L (2018) False-positive Xpert MTB/RIF results in retested patients with previous tuberculosis: frequency, profile, and prospective clinical outcomes. J Clin Microbiol 56(3):01696–01617. 10.1128/jcm PMC5824043 29305538

[38] Balcells ME, Huilcamán M, Peña C (2016) M. tuberculosis DNA detection in nasopharyngeal mucosa can precede tuberculosis development in contacts. Int J Tuberc Lung Dis 20(6):848–852 27155192 10.5588/ijtld.15.0872

[39] Beynon F, Theron G, Respeito D (2018) Correlation of Xpert MTB/RIF with measures to assess Mycobacterium tuberculosis bacillary burden in high HIV burden areas of Southern Africa. Sci Rep 8(1):5201 29581435 10.1038/s41598-018-23066-2 PMC5980110

[40] Yerlikaya S, Chirwa M, Ajide B (2026) Pulmonary Tuberculosis Detection with MiniDock MTB Using Swab Samples. N Engl J Med 394(17):1710–1722 42054680 10.1056/NEJMoa2509761 PMC13132025

[41] de VosM, LeH, MarceloD (2026) Multicountry assessment of tongue swabs for tuberculosis using a common protocol for Xpert MTB/RIF Ultra testing: a prospective diagnostic accuracy study. Lancet Microbe10.1016/j.lanmic.2025.10133042134368

[42] Kumar KM, Borkman A, Kim A (2026) Preferences for tongue swab versus sputum collection for tuberculosis testing: a multi-country survey. Int J tuberculosis lung disease: official J Int Union against Tuberculosis Lung Disease 30(3):134 10.5588/ijtld.25.0457 PMC13245111 41761390

[43] Bezuidenhout C, Long L, Nichols B (2026) Sputum and tongue swab molecular testing for the in-home diagnosis of tuberculosis in unselected household contacts: a cost and cost-effectiveness analysis. Clin Infect Dis 82(1):49–57 40705837 10.1093/cid/ciaf389

